# Psychometric properties of an innovative smartphone application to investigate the daily impact of hypoglycemia in people with type 1 or type 2 diabetes: The Hypo-METRICS app

**DOI:** 10.1371/journal.pone.0283148

**Published:** 2023-03-17

**Authors:** Uffe Søholm, Melanie Broadley, Natalie Zaremba, Patrick Divilly, Giesje Nefs, Jill Carlton, Julia K. Mader, Petra Martina Baumann, Mikel Gomes, Gilberte Martine-Edith, Daniel J. Pollard, Dajana Rath, Simon Heller, Ulrik Pedersen-Bjergaard, Rory J. McCrimmon, Eric Renard, Mark Evans, Bastiaan de Galan, Thomas Forkmann, Stephanie A. Amiel, Christel Hendrieckx, Jane Speight, Pratik Choudhary, Frans Pouwer, on behalf of the Hypo-RESOLVE consortium

**Affiliations:** 1 Medical & Science, Patient Focused Drug Development, Novo Nordisk A/S, Søborg, Denmark; 2 Department of Psychology, University of Southern Denmark, Odense, Denmark; 3 Faculty of Life Sciences and Medicine, Department of Diabetes, School of Cardiovascular Medicine and Sciences, King’s College London, London, United Kingdom; 4 Department of Medical Psychology, Radboud Institute for Health Sciences, Radboud University Medical Centre, Nijmegen, the Netherlands; 5 Department of Medical and Clinical Psychology, Tilburg University, Tilburg, the Netherlands; 6 National Treatment and Research Center for Children, Adolescents and Adults with Type 1 Diabetes, Rotterdam, The Netherlands; 7 School of Health and Related Research (ScHARR), University of Sheffield, Sheffield, United Kingdom; 8 Department of Internal Medicine, Division of Endocrinology and Diabetology, Medical University of Graz, Graz, Austria; 9 Division of Endocrinology & Diabetology, Medical University of Graz, Graz, Austria; 10 Digital Therapeutics, Scientific Modelling, Novo Nordisk A/S, Søborg, Denmark; 11 Department of Clinical Psychology, University of Duisburg-Essen, Essen, Germany; 12 Department of Oncology and Metabolism, University of Sheffield, Sheffield, United Kingdom; 13 Department of Endocrinology and Nephrology, Nordsjællands Hospital Hillerød, Hillerød, Denmark; 14 Institute of Clinical Medicine, University of Copenhagen, Copenhagen, Denmark; 15 Systems Medicine, School of Medicine, University of Dundee, Dundee, United Kingdom; 16 Department of Endocrinology, Diabetes, Nutrition, Montpellier University Hospital, Montpellier, France; 17 Institute of Functional Genomics, University of Montpellier, CNRS, INSERM, Montpellier, France; 18 Welcome Trust-MRC Institute of Metabolic Science and Department of Medicine, University of Cambridge, Cambridge, United Kingdom; 19 Department of Internal Medicine, Radboud University Medical Centre, Nijmegen, the Netherlands; 20 Department of Internal Medicine, Division of Endocrinology and Metabolic Disease, Maastricht University Medical Centre, Maastricht, the Netherlands; 21 CARIM School for Cardiovascular Diseases, Maastricht University, Maastricht, the Netherlands; 22 School of Psychology, Deakin University, Geelong, Australia; 23 The Australian Centre for Behavioural Research in Diabetes, Diabetes Victoria, Melbourne, Australia; 24 Diabetes Research Centre, University of Leicester, Leicester, United Kingdom; 25 Steno Diabetes Center Odense (SDCO), Odense, Denmark; University of Illinois at Urbana-Champaign, UNITED STATES

## Abstract

**Introduction:**

The aim of this study was to determine the acceptability and psychometric properties of the Hypo-METRICS (Hypoglycemia MEasurement, ThResholds and ImpaCtS) application (app): a novel tool designed to assess the direct impact of symptomatic and asymptomatic hypoglycemia on daily functioning in people with insulin-treated diabetes.

**Materials and methods:**

100 adults with type 1 diabetes mellitus (T1DM, n = 64) or insulin-treated type 2 diabetes mellitus (T2DM, n = 36) completed three daily ‘check-ins’ (morning, afternoon and evening) via the Hypo-METRICs app across 10 weeks, to respond to 29 unique questions about their subjective daily functioning. Questions addressed sleep quality, energy level, mood, affect, cognitive functioning, fear of hypoglycemia and hyperglycemia, social functioning, and work/productivity. Completion rates, structural validity, internal consistency, and test-retest reliability were explored. App responses were correlated with validated person-reported outcome measures to investigate convergent (r_s_>±0.3) and divergent (r_s_<±0.3) validity.

**Results:**

Participants’ mean±SD age was 54±16 years, diabetes duration was 23±13 years, and most recent HbA1c was 56.6±9.8 mmol/mol. Participants submitted mean±SD 191±16 out of 210 possible ‘check-ins’ (91%). Structural validity was confirmed with multi-level confirmatory factor analysis showing good model fit on the adjusted model (Comparative Fit Index >0.95, Root-Mean-Square Error of Approximation <0.06, Standardized Root-Mean-square Residual<0.08). Scales had satisfactory internal consistency (all ω≥0.5), and high test-retest reliability (r_s_≥0.7). Convergent and divergent validity were demonstrated for most scales.

**Conclusion:**

High completion rates and satisfactory psychometric properties demonstrated that the Hypo-METRICS app is acceptable to adults with T1DM and T2DM, and a reliable and valid tool to explore the daily impact of hypoglycemia.

## Introduction

Hypoglycemia remains a daily threat for most people with type 1 diabetes (T1DM) or insulin-treated type 2 diabetes (T2DM). Hypoglycemia impacts on many areas of daily life including sleep duration and quality, mood, cognition, and productivity, and can in extreme cases lead to coma or even death [1–5]. Both the experience of hypoglycemia, and living with the risk of hypoglycemia (including prevention and treatment) can present a significant burden for people with diabetes [6].

Previous studies on the impact of hypoglycemia have important limitations. These include recall bias for retrospective measures [7], low ecological validity for hospital-based studies [8], limited insight into the impact of asymptomatic episodes detected by continuous glucose monitoring (CGM) [9], and scarce investigation of impact beyond health status and fear of hypoglycemia [10,11]. The Hypo-METRICS (Hypoglycemia MEasurement, ThResholds and ImpaCtS) study attempts to address these limitations and further our understanding of hypoglycemia in its different forms, i.e. symptomatic and asymptomatic, severe and self-treated, and while awake and during sleep [12]. To capture data temporally closer to the hypoglycemic episodes as they occur in real-life, an ecological momentary assessment smartphone application (Hypo-METRICS app) in which experiences are repeatedly captured in real-time and in a usual environment, has been developed. The app prompts participants (morning, afternoon, and evening) to respond to questions within a few hours of any hypoglycemic episode occurring in their everyday lives.

To investigate whether broad use of the Hypo-METRICS app in research and practice is indicated, this study investigates the app’s acceptability (completion rates) and psychometric characteristics (structural validity, internal consistency reliability, test-retest reliability, convergent and divergent validity). These investigations are essential instrument validation steps, that are required before we can address the Hypo-METRICS study key objectives (outlined in Divilly et al [13]) in future publications.

## Materials and methods

### Study participants & procedure

Hypo-METRICS is part of the EU IMI2-funded Hypo-RESOLVE (Hypoglycemia–Redefining SOLutions for better liVEs) project [12,13], across five European countries (Austria, Denmark, France, the Netherlands, and the United Kingdom). The Hypo-METRICS clinical study has received ethical approval at the lead site from the South Central Oxford B Research Ethics Committee (20/SC/0112) and in the other European countries (Ethikkommission der Medizinischen Universität Graz (Austria), Videnskabsetisk Komite for Region Hovedstaden (Denmark), Comité De Protection Des Personnes SUD Mediterranne IV (France), and Commissie Mensgebonden Onderzoek Regio Arnhem-Nijmegen (the Netherlands)).

Eligible participants were aged 18–85 years, had experienced at least one self-reported hypoglycemic episode in the past 3 months, and were willing to complete the app three times per day and wear a blinded CGM for 10 weeks. Informed written consent was obtained from all participants. Participants from the following three groups were recruited: a) T1DM with intact awareness of hypoglycemia (Gold Score <4 [14]), b) T1DM with impaired awareness of hypoglycemia (Gold score ≥4 [14]), and c) T2DM managed with ≥1 insulin injection per day. The sample for the present study was determined *a priori* and consists of the first 100 participants to complete the Hypo-METRICS study. This sample size was based on guidelines suggesting an item-to-participant ratio of 1:10 [15] for evaluation of structural validity, and on the requirement to confirm the conceptual model fit before analyses could proceed for central study objectives. Before and after the 10-week period, participants completed an online survey (via Qualtrics, Provo, UT) with person-reported outcome measures (PROMs) and demographic and clinical characteristics were collected.

### Study measures

#### The Hypo-METRICS app

The app consists of seven modules with 29 unique questions (see S1 Table), most of which use interval response scales (range 0–10, e.g., “not at all (0)” to “extremely (10)”). The app questions were designed to examine the impact of hypoglycemia on various domains of daily functioning, across three “check-ins”: morning, afternoon, and evening (S1 Table). There was a “Skip question” option for each question. The app was developed in English (UK) and translated into Danish, Dutch, French, and German following the ISPOR guidance [16]. Further details about the app design and development are published elsewhere [17].

#### Additional PROMs

Validated PROMs were included for the purpose of validating the Hypo-METRICS app. Two of these were also completed via the app platform on a weekly basis, while the remaining were completed via Qualtrics (Qualtrics, Provo, UT) at baseline and end of study (see Table 1). PROMs were selected to examine constructs (e.g., ‘mood’ or ‘cognitive functioning’) either similar to those measured by the app for convergent validity, or dissimilar, for divergent validity. Moderate-to-high correlations (r_s_>±0.3) were expected to evidence convergent validity, and low or no correlations (r_s_<±0.3) were expected for divergent validity.

**Table 1:** 

Construct measured	Patient reported outcome measure.
Sleep quality / sleep disturbance	^1^Patient-Reported Outcomes Measurement Information System (PROMIS)—Sleep Disturbance–Short Form 8b [18]
Depression	Patient Health Questionnaire– 9 (PHQ-9) [19]
Anxiety	General Anxiety Disorder-7 (GAD-7) [20]
Vitality	Vitality subscale SF-36 [21]
Cognitive functioning	Perceived Deficit Questionnaire (PDQ-20) [22]
Fear of hypoglycaemia	Hypoglycaemic Fear Survey II (HFS-II) [23]
Diabetes Distress	Problem Areas In Diabetes (PAID-20) [24]
Diabetes specific Quality of life	Dawn Impact of Diabetes Profile (DIDP) [25]
Work and productivity	^1^Work Productivity and Activity Impairment Questionnaire: Specific Health Problem (WPAI:SHP) [26]

^1^These were applied via the Hypo-METRICS app on a weekly basis, while the remaining questionnaires were applied at baseline and follow-up via Qualtrics (Qualtrics, Provo, UT).

#### Demographic & clinical data

At the start of the study, age, gender, employment status, education, medical history, previous episodes of hypoglycemia, HbA1c and method of glucose monitoring, were recorded by the study site personnel and entered into an electronic database (REDCAP, Vanderbilt, USA).

### Statistical analysis

Statistical analyses were conducted with R Studio [27]. Descriptive statistics were used to determine sample characteristics, completion rates, distribution of the data, and floor and ceiling effects. In case of non-normality of question responses, non-parametric tests (Spearman’s rho, r_s_) were applied. Between-person variance was examined using the Intraclass Correlation Coefficient (ICC) [28] and day-to-day variability in scores was examined using the Root Mean Squared Successive Difference (RMSSD) [29]. To examine structural validity, a multi-level confirmatory factor analysis (MCFA) was conducted. The following indices and values were used as indication of good global model fit: comparative fit index (CFI) >0.95, Tucker Lewis index (TLI) >0.95, the Standardized Root-Mean-square Residual (SRMR) <0.08 and Root-Mean-Square Error of Approximation (RMSEA) <0.06 [30,31]. Items were included in the MCFA if they: 1) were asked every day irrespective of whether the participant experienced a hypoglycemic episode, and 2) were not part of the work and productivity module of the app, where most items are relevant only to participants engaged in paid work. With 100 participants and a maximum of 10 unique items per check-in, a 1:10 item to participant ratio was considered acceptable for conducting factor analysis [15]. Internal consistency reliability of the scales were calculated with use of McDonald’s ω with scores r_s_>0.7 considered to indicate satisfactory internal consistency [32]. Question responses were analyzed according to check-in time: morning, afternoon or evening. These were analyzed separately to account for potential variation in latent factors at different timepoints of the day (e.g., fear of hypoglycemia in the daytime could be different to fear of hypoglycemia in the night-time). Finally, convergent and divergent validity were investigated by correlating question and scale scores with validated PROMs and participant characteristics. For more details on statistical analyses see (S1 Text).

## Results

### Participant characteristics

The first 100 participants who completed the Hypo-METRICS study included 64 adults with T1DM and 36 adults with T2DM, from the United Kingdom (57%) or the Netherlands (43%). Table 2 presents the participant characteristics. Overall, participants’ [M±SD] age was 54±16 years, diabetes duration was 23±13 years and most recent HbA_1c_ was 56.6±9.8mmol/mol. For T1DM and T2DM respectively, 22% and 17% had impaired awareness of hypoglycemia (Gold score ≥4), 13% and 8% had moderate-severe anxiety, and 13% and 17% had moderate-severe depression as determined by the GAD-7 and PHQ-9.

**Table 2:** Participants’ demographic, clinical and psychosocial characteristics at baseline (N = 100).

Demographic and clinical characteristics	Type 1 diabetes (n = 64)	Type 2 diabetes (n = 36)
Age,years (range)***	47 ± 15 (21–80)	66 ± 9 (43–79)
**Women**	27 (42%)	17 (47%)
Employment***		
Full time employment	32 (50%)	6 (17%)
Part time employment	12 (19%)	3 (8.3%)
Full time education	2 (3.1%)	0 (0%)
Unemployed but actively looking for work	5 (7.8%)	0 (0%)
Unemployed but not actively looking for work	2 (3.1%)	4 (11%)
Retired	11 (17%)	23 (64%)
Highest level of education achieved***		
Primary school	0 (0%)	1 (2.8%)
Secondary school / high school	14 (22%)	14 (39%)
College / undergraduate degree	29 (45%)	11 (31%)
Post graduate degree	18 (28%)	1 (2.8%)
Other	3 (4.7%)	9 (25%)
**Diabetes duration (years)**	22.95 ± 14.82	22.06 ± 9.71
**HbA1c (baseline)**		
%	7.32 ± 0.84	7.35 ± 1.01
Mmol/mol	56.53 ± 9.18	56.85 ± 11.04
Percentage time below 70mg/dL***	6.10 (4.58)	2.06 (2.01)
**Microvascular complications, any**	18 (28%)	17 (47%)
Microvascular complications, any*	10 (16%)	13 (36%)
**Usual method of glucose monitoring**		
Capillary glucose monitoring only (fingerprick)*	20 (31%)	19 (53%)
Continuous glucose monitoring without alerts**	48 (75%)	17 (47%)
Continuous glucose monitoring with alerts	1 (1.6%)	0 (0%)
Country***		
United Kingdom	45 (70%)	12 (33%)
The Netherlands	19 (30%)	24 (67%)
**Gold score**		
Impaired awareness (≥4)	14 (22%)	6 (17%)
Intact awareness (<4)	49 (78%)	30 (83%)
Missing	1	0
**Psychosocial characteristics**		
Anxiety symptoms (GAD-7) No anxiety (<5)	46 (73%)	28 (78%)
Mild anxiety (5–10)	9 (14%)	5 (14%)
Moderate-Severe anxiety (≥10)	8 (13%)	3 (8.3%)
Missing	1	0
**Depression symptoms (PHQ-9)**		
No depression (<5)	44 (70%)	27 (75%)
Mild depression (5–10)	11 (17%)	3 (8.3%)
Moderate-Severe depression (≥10)	8 (13%)	6 (17%)
Missing	1	0
**Cognitive functioning (PDQ-20)^1^**	18.62 ± 13.44 (n = 63)	16.51 ± 10.72
**Diabetes-specific quality of life (DIDP)^2^**		
Composite score	4.48 ± 0.81	4.39 ± 0.83
Percentage score	49.72 ± 11.63	48.44 ± 11.88
Missing	1	0
Fear of hypoglycemia (HFS-II total)^3^ **	32.98 ± 22.17 (n = 63)	21.22 ± 14.72
**Sleep-quality score (T-score PROMIS week 10)^4^**	49.77 ± 8.85 (n = 58)	50.49 ± 8.68
**Vitality (SF-36 vitality subscale mean)^5^**	3.35 ± 0.83 (n = 63)	3.37 ± 0.63
**Diabetes distress (PAID total)^6^**	21.08 ± 17.27 (n = 60)	17.89 ± 15.81 (n = 63)
Below 40	54 (86%)	33 (92%)
Above 40	9 (14%)	3 (8.3%)
Missing	1	0

Data are mean ± SD or n (%).

^a^igher scores indicate greater perceived cognitive difficulties.

^2^Higher scores indicate greater negative impact across global life dimensions (possible ranges from 1–7 and 1–100 for composite and percentage scores respectively).

^3^Higher scores indicate higher fear of hypoglycemia.

^4^Higher scores indicate higher sleep disturbance (lower sleep quality).

^54^Higher scores indicate higher fatigue (less energetic).

^6^PAID scores above 40 indicate severe diabetes distress.

Test of difference between T1DM and T2DM group: *p<0.05 **p<0.01 ***p<0.001.

### Completion rates and acceptability

Participants completed 191±16 of 210 possible check-ins, a completion rate of 91%. Slight differences in completion between the morning, afternoon and evening check-ins were observed with completion rates of 90%, 89% and 94%, respectively. When a check-in was submitted, all questions (except the work/productivity questions) were completed more than 99% of the time (Table 3). One question (“*How well did you get along with other people today*?”), was skipped marginally more frequently (0.9%) than other questions. Questions in the work and productivity section were frequently skipped (range: 36–47%). Inspection of histograms revealed floor effects as indicated by more than 15% (range: 15–28%) of responses on the lowest score for the four negatively phrased questions (“*How anxious do you feel right now*?”, “*How irritable do you feel right now*?”, “*How worried are you about having a hypo later today/while asleep*?” and “*How worried are you about having high blood glucose later today/while asleep*?” across the three check-ins).

**Table 3:** Latent factors and descriptive statistics of questions.

Latent factors		Morning questions	Mean	SD	Skipped n (%)^1^	ICC^2^	RMSSD^3^
**Sleep quality**		1. How did you sleep?	7.03	1.79	8 (0.13)	0.43	1.76 ± 0.74 (0)
		2. When you woke up, how did you feel?	6.45	2.02	7 (0.11)	0.63	1.62 ± 0.67 (0)
**Overall mood**		3. How is your mood right now?	7.06	1.65	10 (0.16)	0.56	1.41 ± 0.62 (1)
**Negative affect**		4. How irritable do you feel right now?*	8.20	1.89	12 (0.19)	0.58	1.51 ± 0.85 (2)
		5. How anxious do you feel right now?*	8.23	2.01	13 (0.21)	0.64	1.34 ± 1.00 (13)
**Energy level**		6. How is your energy level right now?	6.61	1.86	7 (0.11)	0.64	1.43 ± 0.66 (2)
**Cognitive functioning**		7. How alert do you feel right now?	6.87	1.79	8 (0.13)	0.64	1.33 ± 0.7 (2)
		8. How well are you able to concentrate right now?	7.15	1.63	9 (0.14)	0.65	1.22 ± 0.58 (1)
**Fear of hypoglycemia**		9. How worried are you about having a hypo later today?*	7.59	2.30	5 (0.08)	0.83	1.06 ± 0.80 (14)
**Fear of hyperglycemia**		10. How worried are you about having high blood glucose later today?*	6.64	2.84	3 (0.05)	0.85	1.29 ± 0.89 (10)
		**Afternoon questions**	**Mean**	**SD**	**Skipped n (%)** ^**1**^	**ICC** ^**2**^	**RMSSD** ^**3**^
**Overall mood**		1. How is your mood right now?	7.33	1.55	4 (0.06)	0.52	1.37 ± 0.66 (1)
**Negative affect**		2. How irritable do you feel right now?*	8.21	1.88	6 (0.10)	0.54	1.59 ± 0.85 (2)
		3. How anxious do you feel right now?*	8.25	2.01	10 (0.16)	0.58	1.52 ± 1.02 (10)
**Energy level**		4. How is your energy level right now?	6.72	1.76	6 (0.10)	0.57	1.47 ± 0.68 (1)
**Cognitive functioning**		5. How alert do you feel right now?	7.04	1.68	5 (0.08)	0.59	1.35 ± 0.68 (1)
		6. How well are you able to concentrate right now?	7.23	1.63	3 (0.05)	0.60	1.29 ± 0.64 (1)
**Fear of hypoglycemia**		7. How worried are you about having a hypo later today?*	7.61	2.34	2 (0.03)	0.82	1.18 ± 0.80 (9)
**Fear of hyperglycemia**		8. How worried are you about having high blood glucose later today?*	6.61	2.88	1 (0.02)	0.85	1.30 ± 0.74 (7)
		**Evening questions**	**Mean**	**SD**	**Skipped n (%)** ^**1**^	**ICC** ^**2**^	**RMSSD** ^**3**^
**Overall mood**		1. How is your mood right now?	7.14	1.60	9 (0.14)	0.52	1.39 ± 0.69 (1)
**Negative affect**		2. How irritable do you feel right now?*	8.20	1.90	8 (0.12)	0.55	1.56 ± 0.92 (4)
		3. How anxious do you feel right now?*	8.26	1.98	10 (0.15)	0.61	1.47 ± 0.95 (9)
**Energy level**		4. How is your energy level right now?	6.23	1.83	5 (0.08)	0.61	1.47 ± 0.71 (1)
**Cognitive functioning**		5. How alert do you feel right now?	6.54	1.82	4 (0.06)	0.64	1.36 ± 0.76 (1)
		6. How well are you able to concentrate right now?	6.97	1.70	4 (0.06)	0.64	1.29 ± 0.67 (2)
**Memory (today)**		7. How easy was it for you to remember things today?	7.43	1.56	6 (0.09)	0.66	1.15 ± 0.62 (1)
**Fear of hypoglycemia while asleep**		8. How worried are you about having a hypo while asleep?*	7.38	2.54	1 (0.02)	0.83	1.20 ± 0.87 (11)
**Fear of** **Hyperglycemia while asleep**		9. How worried are you about having high blood glucose while asleep?*	6.60	2.88	1 (0.02)	0.84	1.31 ± 0.97 (9)
**Social functioning**		10. How well did you get along with other people today?	7.80	1.47	58 (0.88)	0.62	1.10 ± 0.61 (2)
		**Work and productivity questions**	**Mean**	**SD**	**Skipped n (%)** ^**1**^	**ICC** ^**2**^	**RMSSD** ^**3**^
**Work and productivity^4^**		1. How many hours did you work today?	4.25	3.83	2748 (41.72)	0.25	3.01 ± 2.31 (22)
		2. How many hours did you miss from work for ANY reason today? [this includes health issues, vacation, holiday, etc.]	0.51	1.77	2899 (44.02)	0.15	0.99 ± 1.50 (40)
		3. How many hours did you miss from activities other than work today for ANY reason (e.g. study, housework, shopping, family or leisure activities)?	0.33	1.16	2348 (35.65)	0.51	0.68 ± 0.87 (33)
		4. How productive were you while working today?	7.07	2.10	3079 (46.75)	0.45	1.21 ± 1.11 (22)

^**1**^**Skipped:** The participant has submitted the check-in but skipped the individual question (by selecting “Skip question”).

^**2**^**ICC:** Intraclass correlation; proportion of variance attributed to between-person variance (relative to total- person variance).

^**3**^**RMSSD:** Root Mean Squared Successive Difference. Values indicate: Mean ± SD (n with no variability on the question).

^4^The four work and productivity questions are conceptually alike but are not intended to be combined into one scale, but rather analysed as four separate scales. For question 1, 2 and 3, hours >24 hours (invalid responses) were excluded.

*Reverse-scored questions: Higher scores indicate better daily functioning.

Range for all questions was 0–10 in the morning, afternoon and evening check-in. Range for work and productivity questions was 0–24 hours for questions 1 and 2, 0–20 hours for question 3 and 0–10 for question 4.

### Structural validity and internal consistency reliability

Based on kurtosis values and histograms, data were considered non-normally distributed. The ICC ranged from 0.15 to 0.85 and all but two questions (both relating to sleep) included at least one participant with zero variability across the study period (RMSSD = 0) (Table 3). Exploring the data further revealed that one participant had zero variability across all questions (with the exception of the two sleep questions), but their data were still included in further analyses as removing it did not change conclusions. Of the non-work related questions, the negatively phrased questions had the least day-to-day variability. Inter-question correlations were acceptable (r_s_ = 0.20–0.81) and multicollinearity was absent (determinant range 0.000922–0.0134 across the three check-ins). Kaiser-Meyer-Olkin values ranged from 0.76–0.93 across all questions suggesting good factorability.

Applying the five-step approach by Huang [33] to the morning check-in suggested that a MCFA was appropriate (see S2 Table). The first model, based on the conceptual framework (model A in S3 Table), had good model fit on several model fit indices, however, at the between-person level, the SRMR>0.8 (morning and afternoon) indicated that model fit could be further improved. Inspection of correlation residuals (as well as modification indices) showed a large residual between the irritability and anxiety questions at both levels, suggesting that a relationship between these two was not captured in the original model. Combining the two into one scale improved (i.e., decreased) the between-SRMR model fit parameter across all three check-ins (model B in S3 Table). The new scale was labelled ‘Negative affect’ (and the single-question mood scale was labelled ‘Overall mood’). Inspection of the internal consistencies (ω) of model B showed that ω was low for the fear of hypo-/hyperglycemia factor on the within-person level, with values ranging between 0.19–0.30. Therefore, it was decided not to combine these two questions in the same factor. This led to the final model (model C in S3 Table), which showed good model fit on several fit indices across the check-ins (CFI>0.95, RMSEA<0.06, SRMR<0.08). In model C, standardized between-person factor loadings for all questions were >0.7 (Table 4), indicating that factors explained the grouping of the questions well, while at the within-person level, the majority of loadings for the two-question ‘Negative affect’ scale (across the three check-ins) were <0.7. There was a similar pattern across other scales, with satisfactory internal consistency (ω>0.7) at the between-person level but slightly lower (ω>0.5) for the ‘Negative affect’ and ‘Cognitive functioning’ scales at the within-person level (Table 4).

**Table 4:** Standardized factor loadings and internal consistency measures (adjusted model C).

Latent variable		Std. factor loadings		Internal Consistency (ω)	
		Within	Between	Within	Between
	**Morning questions**				
**Sleep quality**	How did you sleep?	0.74	0.87	0.77	0.91
	When you woke up, how did you feel?	0.85	0.94		
**Overall mood**	How is your mood right now?	1.00	1.00	NA	NA
**Negative affect**	How anxious do you feel right now?	0.55	0.92	0.55	0.93
	How irritable do you feel right now?	0.68	0.95		
**Energy level**	How is your energy level right now?	1.00	1.00	NA	NA
**Cognitive functioning**	How alert do you feel right now?	0.80	0.93	0.78	0.95
	How well are you able to concentrate right now?	0.80	0.98		
**Fear of hypoglycemia**	How worried are you about having a hypo later today?	1.00	1.00	NA	NA
**Fear of hyperglycemia**	How worried are you about having high blood glucose later today?	1.00	1.00	NA	NA
	**Afternoon questions**				
**Overall mood**	How is your mood right now?	1.00	1.00	NA	NA
**Negative affect**	How anxious do you feel right now?	0.52	0.94	0.53	0.93
	How irritable do you feel right now?*	0.68	0.92		
**Energy level**	How is your energy level right now?	1.00	1.00	NA	NA
**Cognitive functioning**	How alert do you feel right now?	0.80	0.89	0.75	0.95
	How well are you able to concentrate right now?	0.74	1.01		
**Fear of hypoglycemia**	How worried are you about having a hypo later today?	1.00	1.00	NA	NA
**Fear of hyperglycemia**	How worried are you about having high blood glucose later today?	1.00	1.00	NA	NA
	**Evening questions**				
**Overall mood**	How is your mood right now?	1.00	1.00	NA	NA
**Negative affect**	How anxious do you feel right now?	0.52	0.91	0.54	0.93
	How irritable do you feel right now?	0.70	0.96		
**Energy level**	How is your energy level right now?	1.00	1.00	NA	NA
**Cognitive functioning**	How alert do you feel right now?	0.71	0.86	0.66	0.92
	How well are you able to concentrate right now?	0.70	0.99		
**Memory (today)**	How easy was it for you to remember things today?	1.00	1.00	NA	NA
**Fear of hypoglycemia while asleep**	How worried are you about having a hypo while asleep?	1.00	1.00	NA	NA
**Fear of hyperglycemia while asleep**	How worried are you about having high blood glucose while asleep?	1.00	1.00	NA	NA
**Social functioning**	How well did you get along with other people today?	1.00	1.00	NA	NA

NA: Not applicable.

### Test-retest reliability, convergent and divergent validity

**Fig 1:**
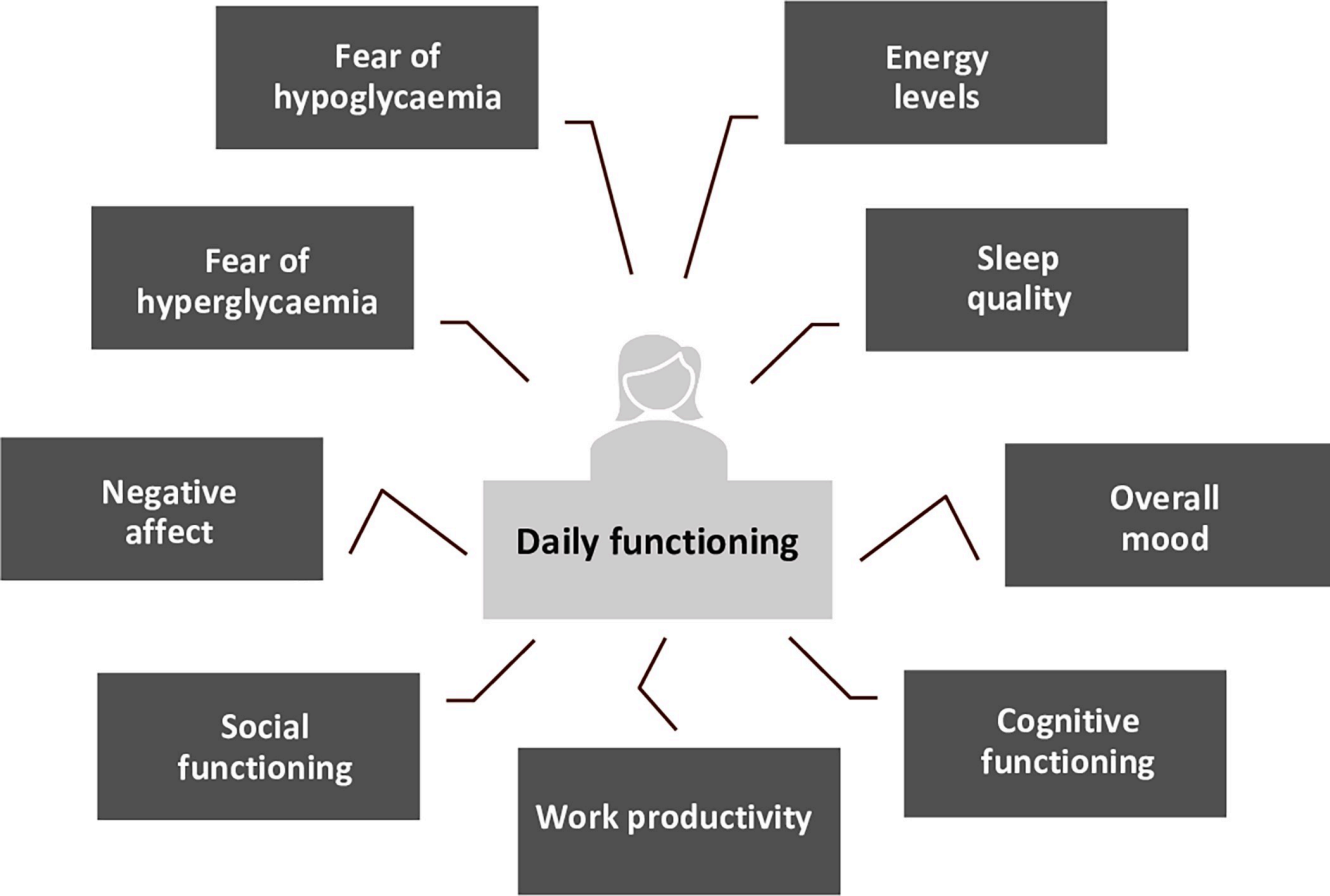
The overall domains of daily functioning assessed by the Hypo-METRICS app.

Test-retest reliability and convergent and divergent validity were explored for each scale from model C. High test-retest correlations (r = 0.76–0.94) were found across all two-questions and single-question scales (Table 5). For convergent validity, the hypothesized pattern of correlations with PROMs was largely supported (Table 5), except for ‘Energy level’ (morning), ‘Cognitive functioning’ (morning, afternoon), ‘Memory’ (evening), ‘Fear of hyperglycemia while asleep’ (evening) and ‘Social functioning’ (evening). On the other hand, the PROMs measuring vitality and cognitive functioning did correlate highest with the respective app scales (‘Energy level’ and ‘Cognitive functioning’) compared to all other app scales (i.e., when reading vertically down the Table 5 columns). Divergent validity was evidenced by the lowest correlations between all app scales and the ‘Financial situation (DIDP)’ question, ‘HbA_1c_’ and ‘Diabetes duration’ (included solely for expected low correlations). However, many of the correlations in the remaining dark grey boxes in Table 5 (indicating other correlations that were expected to be low) were above r_s_>±0.3 (e.g., in the morning between ‘Overall mood’ and ‘Cognitive functioning’, ‘Negative affect’ and ‘Vitality’). The validated weekly work-productivity questionnaire (see S4 Table), showed a strong correlation (r>0.5) with ‘number of hours worked’, a moderate correlation (r>0.3) with ‘productivity’ questions, and low correlations (r<0.3) with hours missed from work and activities other than work on the app. Fig 1 provides an overview of the overall domains of daily functioning that the Hypo-METRICS app is believed to assess based on the psychometric analyses performed.

**Table 5:** Test-retest and convergent and divergent validity of the Hypo-METRICS app scales and questions.

	Test-retest^1^	Sleep quality (PROMIS)	Depression (PHQ-9)	Anxiety (GAD-7)	Vitality (SF-36)	Cognitive functioning (PDQ-20)	Fear of hypoglycemia (HFS-II)	Diabetes distress (PAID-20)^8^		Financial situation (DIDP question)^8^		HbA1C (mmol/mol)^8^	Diabetes duration^8^
**Morning**													
Sleep quality	0.83	**-0.68**	-0.62	-0.51	0.52	-0.46	-0.29*	-0.35*		-0.18^ns^		-0.11^ns^	0.07^ns^
Overall mood	0.81	-0.56	**-0.68**	**-0.60**	0.55	-0.53	-0.28^ns^	-0.41		-0.21^ns^		-0.12^ns^	0.01^ns^
Negative affect	0.83	-0.47	-0.63	**-0.70**	0.50	-0.52	-0.41	-0.50		-0.11^ns^		-0.19^ns^	0.04^ns^
Energy level	0.86	-0.51	-0.68	-0.54	0.65	-0.53	-0.34*	-0.40		-0.20^s^		-0.11^ns^	-0.03^ns^
Cognitive functioning	0.88	-0.45	-0.65	-0.59	0.55	-0.57	-0.21^ns^	-0.39*		-0.18^ns^		-0.10^ns^	0.06^ns^
Fear of hypoglycemia	0.94	-0.33*	-0.36*	-0.40	0.27^ns^	-0.28*	**-0.60**	-0.51		-0.15^ns^		-0.04^ns^	0.16^ns^
Fear of hyperglycemia	0.93	-0.34*	-0.38*	-0.36*	0.36*	-0.37*	-0.53	-0.48^**2**^	**-0.49** ^**3**^	-0.18^ns^		-0.12^ns^	0.05^ns^
**Afternoon**													
Overall mood	0.82	-0.54	**-0.60**	**-0.55**	0.51	-0.46	-0.25^ns^	-0.39*		-0.17^ns^		-0.22^ns^	-0.02^ns^
Negative affect	0.86	-0.47	-0.62	**-0.66**	0.49	-0.51	-0.41	-0.53		-0.06^ns^		-0.14^ns^	0.03^ns^
Energy level	0.85	-0.49	-0.58	-0.46	**0.60**	-0.53	-0.35*	-0.34*		-0.16^ns^		-0.16^ns^	0.03^ns^
Cognitive functioning	0.85	-0.42	-0.58	-0.53	0.51	-0.54	-0.18^ns^	-0.37*		-0.13^ns^		-0.09^ns^	0.07^ns^
Fear of hypoglycemia	0.93	-0.38*	-0.40	-0.43	0.29*	-0.30*	**-0.57**	-0.51		-0.13^ns^		-0.04^ns^	0.21^ns^
Fear of hyperglycemia	0.91	-0.35*	-0.40	-0.36*	0.37*	-0.39*	-0.53	-0.49^**2**^	**-0.49** ^**3**^	-0.17^ns^		-0.11^ns^	0.07^ns^
**Evening**													
Overall mood	0.76	-0.46	**-0.61**	**-0.58**	0.53	-0.51	-0.25^ns^	-0.40*		-0.25^ns^		-0.17^ns^	0.03^ns^
Negative affect	0.85	-0.45	-0.62	**-0.70**	0.50	-0.53	-0.44	-0.55		-0.13^ns^		-0.19^ns^	0.06^ns^
Energy level	0.83	-0.41	-0.57	-0.48	**0.61**	-0.57	-0.36*	-0.38*		-0.24^ns^		-0.13^ns^	0.05^ns^
Cognitive functioning	0.88	-0.36*	-0.55	-0.56	0.54	**-0.61**	-0.23^ns^	-0.34^ns^		-0.20^ns^		-0.06^ns^	0.07^ns^
Memory	0.81	-0.37*	-0.49	-0.50	0.43	-0.49	-0.15^ns^	-0.29^ns^		-0.18^ns^		-0.01^ns^	0.06^ns^
Fear of hypoglycemia while asleep	0.90	-0.33*	-0.41	-0.44	0.25^ns^	-0.32^ns^	**-0.51**	-0.43		-0.15^ns^		-0.06^ns^	0.17^ns^
Fear of hyperglycemia while asleep	0.92	-0.35*	-0.40	-0.39*	0.36*	-0.39*	-0.53	-0.46^2^	-0.47^3^	-0.16^ns^		-0.13^ns^	0.05^ns^
Social functioning	0.82	-0.32*	-0.42	-0.50	0.38*	-0.44	-0.20^ns^	-0.43^4^	-0.18^5 ns^	-0.17^6 ns^	-0.22^7 ns^	-0.10^ns^	0.14^ns^

Given that PROMs typically require respondents to reflect over a given period of time (e.g., ‘past seven days’), daily app scores were averaged over a period of time corresponding to the PROM’s recall period. For example, if the PROM’s recall period was the ‘past seven days’, then the corresponding app scores were averaged across the final seven days of the study period and correlated with the PROM score.

Hypotheses were made for each row (i.e., each app question/scale). In each row, the white cell indicates the correlation that was expected to be highest and the darkest cells indicate the correlations that were expected to be lowest for that question/scale. The lighter grey shading indicates correlations that were expected to fall between the highest and the lowest categories. For example, it was hypothesized that the “energy level” scale would correlate highest with the SF-36 vitality scale, followed by PROMs of related concepts (sleep, depression, anxiety, cognitive function), followed by less conceptually related concepts (diabetes distress, fear of hypoglycemia, financial situation, HbA1c, and diabetes duration).

^1^Test-retest reliability was explored by correlating (Spearman’s rho) average scores on each scale from week 3 (test condition) with average scores from week 8 (re-test). Week 3 was selected to allow a two-week run-in phase for participants to familiarize themselves with the app, and week 8 was select to allow for an inter-test interval similar to other studies [34]. Correlations r>0.7 were considered suitable for demonstrating test-retest reliability.

^2^First number represents correlations with the PAID total score.

^3^Second number represents correlations with the PAID question “*Worrying about the future and the possibility of serious complications*?”

^4^First number represents correlations with the PAID total score.

^5^Second number represents correlations with the PAID question “*Uncomfortable social situations related to your diabetes care (e*.*g*., *people telling you what to eat)*?*”*.

^6^First number represents correlations with the DIDP question “*Your financial situation*”.

^7^Second number represents correlations with the DIDP question “*Your relationship with your family*, *friends and peers*?”.

^8^For these PROMs, there were no specified recall periods, so the average app scores from the last seven days of the study period were used.

Convergent validity was supported if Spearman correlations (between app scales and PROMs) were strong (rs>±0.5) or moderate (rs>±0.3), and divergent validity was supported if they were low (rs<±0.3). ‘Financial situation (DIDP question)’, ‘HbA1c (mmol/mol)’ and ‘Diabetes duration’ were included for exploring divergent validity.

Colours represent hypothesized ranking of correlations

Highest and at least r_s_ > ±0.3.

Medium.

Lowest.

Numbers in bold represent correlations where hypothesized highest correlations were confirmed.

All correlations are significant at a *p* < 0.001, unless otherwise noted: *p<0.05, ns = not significant *p* > 0.05.

Higher scores on all the Hypo-METRICS app scales indicate ‘better’ daily functioning.

## Discussion

This study examined the acceptability and psychometric properties of an innovative smartphone app (Hypo-METRICS): results of the present study support its use as an innovative research tool to determine the impact of hypoglycemia on daily functioning among adults with T1DM or T2DM using insulin. Average completion rates were high and the percentage of skipped questions low. The Hypo-METRICS scales had satisfactory model fit (demonstrated by a MCFA and overall satisfactory ω values), high test-retest reliability and satisfactory convergent and divergent validity. Overall, these findings indicate that the novel Hypo-METRICS app is both valid and reliable for assessing the impact of hypoglycemia on daily functioning in research, with high ecological validity and low recall bias.

The high completion rates suggest that the Hypo-METRICS app is an acceptable instrument for assessments of daily functioning by people with T1DM and insulin-treated T2DM, up to three times per day, seven days per week for up to 10 weeks. All three check-ins were similarly acceptable, which may be attributed to the broad/flexible timeframes and that participants could select a convenient time for app completion. The low percentage of skipped questions (for the non-work-related questions) indicates that questions were generally applicable for most participants. The non-work-related question that was skipped the most was the ‘Social functioning’ question, which could be explained by the context of the COVID-19 pandemic (i.e., data collection occurred during a period of pandemic restrictions on social gatherings). The high percentage of work and productivity questions skipped was expected, as participants were instructed to skip these if they did not have paid employment or if it did not concern a workday. However, the question “*How many hours did you miss from activities other than work today for ANY reason*” does not require the participant to have a paid job to respond to, and the high skip rate could suggest that participants found the question difficult to respond to, difficult to understand, irrelevant, or poorly explained.

At a question-level, the ICC values show that most questions, in particular those focused on worries about hypoglycemia and hyperglycemia, have greater variability between than within individuals. Further, RMSSD values show that for some participants and some questions (particularly negatively-worded and work-related questions), there was no day-to-day variability in responses across the 70-day study period. This may suggest stability in the construct or in sample characteristics (e.g., low baseline depression/anxiety symptoms) and is supported by the floor effects on negatively-worded questions (e.g., the “*How irritable do you feel right now*?”). Alternative explanations could be that the questions were not capable of capturing variability in the construct in this group of participants, or that variability only occurs within days and not between days. The floor effects are not considered problematic, since it is not desirable, or possible, to reach lower scores than ‘not at all’, and most participants had variable responses to these questions over time. The low variability for some questions may also indicate ‘automatic’ or ‘habitual’ responding’, wherein participants select the same responses when presented with the same questions in the same order multiple times [35]. Future studies could explore if question randomization at each check-in would produce different results. The full range on 0–10 scales were used for all app scales, suggesting that the 11-point length was appropriate, however additional work needs to explore minimal important changes on the scales [36].

The structural validity of the app scales was examined using a MCFA. Model C showed good model fit except on the TLI and Chi-square parameters. TLI values were >0.9, which has been considered an acceptable level [37]. The Chi-square test has been argued to provide an unrealistic null-hypothesis and the value is heavily influenced by sample size; therefore, it was considered less important in model selection [30]. The two adjustments made to the original model (model A) were supported by theory. The first adjustment was to move the ‘Irritability’ question (originally paired with ‘Overall mood’), to form a two-question ‘Negative affect’ scale with the ‘Anxiety’ question. As irritability is a facet of mood [38], it was originally paired with mood. However, irritability and anxiety are closely related as they are both aspects of negative emotionality [39]. This latter pairing was better supported by the data. The second adjustment was to separate the ‘Fear of hypoglycemia’ and ‘Fear of hyperglycemia’ questions from an original two-question scale into two, single-question scores. Although these two constructs have previously been found to be significantly correlated [40], the low internal consistency suggests that these did not covary in the current dataset. Further, participants were, on average, more worried about ‘highs’ than ‘lows’, which has been observed clinically and elsewhere [24]. An alternative explanation could be that the variance for the two questions generally was too low to allow them to covary and correlate.

Internal consistency of all app scales was satisfactory (ω >0.7) at the between-person level, but not at the within-person level for ‘Negative affect’ (across all three check-ins) and ‘Cognitive functioning’ (evening check-in). Internal consistency is highly dependent on number of questions in the scale [15], and similar within-person ω-values for two-question scales have been reported in other EMA studies and found acceptable [41,42]. Low ω-values could also reflect greater question heterogeneity than in other pairs of questions, so for analysis at the within-person level only (e.g. N = 1 studies), researchers could consider analyzing single questions rather than scales [43].

EMA methods allow an exploration of the variation in outcomes from timepoint to timepoint. Expecting perfect test-retest reliability (correlations) between assessments contradicts the general assumption of the method [42]. However, if comparing aggregated data (e.g., averaged over a longer time period), representing a person’s traits or general pattern of responding, one could expect more persistent scores across time [42]. This approach has been used in other EMA studies. For example, Csikszentmihalyi et al reported that mean scores on variables measuring affect from the first part of a week correlated highly (r = 0.74) with scores from the second half of the week [42]. The aggregated Hypo-METRICS app scores showed high test-retest reliability, with correlations ranging from r_s_ = 0.76 (for the one-question ‘Overall mood’ scale in the evening) to r_s_>0.9 (for the ‘Fear of hypoglycemia’ and ‘Fear of hyperglycemia’ single questions). These findings suggest reasonable consistency, across a few weeks, in the average scores on the measured constructs.

The correlations between the app scales and validated PROMs overall showed satisfactory convergent validity. The majority of the hypothesized highest correlations (indicative of convergent validity) and lowest correlations (indicative of divergent validity) were confirmed, although some of the hypothesized lowest correlations (e.g. for the ‘Social functioning’ app scale and ‘Anxiety (GAD-7)” PROM) were higher than anticipated. Correlations between app scales (aggregated over periods of 1–4 weeks) and validated PROMs were, in some cases, high (r_s_ up to -0.70). However, it is important to note that no collinearity was present, suggesting that the app is not a redundant measure. Further, EMA offers advantages over retrospective questionnaires: it captures variation in the outcomes over time, and it allows assessment of the direct impact of events (here, episodes of hypoglycemia) on the outcomes. All app scales correlated highly with several PROMs, which was expected as previous studies have shown associations between symptoms of anxiety and depression [44], sleep and mood [45], and depressive symptoms and cognitive functioning [46]. However, some expected correlations were not confirmed in this dataset. A moderate-to-strong correlation was expected, but not confirmed, between the ‘Social functioning’ question in the app and the single questions on the DIDP and PAID scales, which refer to ‘relationships with others’ and ‘uncomfortable social situations’, respectively. However, unlike the PAID and DIDP questions, the ‘Social functioning’ question has no attribution to diabetes/hypoglycemia and is focused on a single day, which may explain the low correlations. Instead, the ‘Social functioning’ question correlated highly with the general anxiety questionnaire. Previous qualitative research has shown that anxieties about unpredictable hypoglycemic episodes limit social activities [47], which supports the strong link between social functioning and anxiety seen here. Further work is required to establish the convergent validity of this question. Future research could also explore the meaning of this question from the perspective of the person with diabetes, using cognitive debriefing.

Evidence for convergent validity of work and productivity questions was mixed. High correlations on ‘number of hours worked’ and moderate correlations on ‘productivity’ questions suggest minimal recall bias on these questions when asked retrospectively for the previous seven days. Hours missed from work and ‘activities other than work’ showed very low correlations between daily and weekly measures, suggesting high recall bias in the PROM or that the questions in the app and the PROM were capturing different information.

A strength of this study is its innovative character, including use of advanced statistical methods suitable to explore the psychometric properties of an app for ecological momentary assessments. Factor analyses are often conducted on cross-sectional data, but when data are clustered with repeated measures per participant, use of standard techniques would violate a general assumption of independency between observations [33,48]. MCFA specifically enables a between- and within-person model to run simultaneously and make it possible for the factor structure to vary across these levels [33]. Another strength of this study was the use of several validated PROMs to examine convergent and divergent validity, and the use of approximately matched time periods for correlations between short-form measures (app scales) and long-form measures (PROMs). Furthermore, psychometric properties were able to be confirmed in the first 100 participants of Hypo-METRICS: a sample including people with T1DM and T2DM, with a balanced gender distribution, and varied methods of glucose monitoring and levels of awareness of hypoglycemia. This was an optimal sample, as it was a balance of being large enough to conduct the planned analyses, while having data collection completed early enough to conduct essential analyses determining if the app was ‘fit for purpose’ prior to analyses of central study objectives. The data were collected in “real” everyday life settings, thereby improving ecological validity with reduced recall burden.

A limitation of this study is that due to the substantial requirements of participants in the Hypo-METRICS study, the sample may reflect a highly motivated and relatively “high functioning” group [49]. Thus, the acceptability of the app needs to be explored in other samples, and/or by use of qualitative methods. Another potential limitation is that, despite participants receiving notifications for each check-in at certain times, there were wide time-intervals (six hours) in which participants could submit the check-ins. Allowing participants to complete check-ins at the most convenient time likely increased engagement with the app but may have biased responses towards more positive daily functioning. Future studies could explore how shorter time-intervals would impact on both completion rates and daily functioning scores.

This study investigated the psychometric properties of the majority of the Hypo-METRICS app questions in the three daily check-ins. However, there are some hypoglycemia-specific questions in the app that were not explored here, as these are not asked at each check-in but only if a hypoglycemic episode was reported (i.e., much higher percentage of missing data must be anticipated for these). Additional work is needed to determine the acceptability and psychometric properties of these questions. Similarly, this study should be replicated in independent samples with diverse characteristics (e.g., ethnic, socio-economic, and health-related). Further research is also needed to fully understand respondents’ completion patterns, including potential predictors of completion. Qualitative research would enable a subjective evaluation of the app completion, including the perceived value and/or burden of using the app across many weeks and ways to improve user experience. Qualitative research would further allow for additional investigation of the content validity (relevance, comprehensiveness and comprehensibility) of the app questions. Future versions of the app could be automated and include conversational agents that, by combining CGM data with daily functioning data from the app, could deliver daily guidance on how to optimize treatment plans and/or improve quality of life.

Overall, these findings show that the Hypo-METRICS app is an acceptable, valid, and reliable tool for research to advance knowledge of how hypoglycemia impacts on daily functioning. In addition to its potential in research, the app may have utility in clinical practice to enhance personalized treatment and care for people with diabetes.

## Supporting information

S1 Table The original conceptual framework of the 29 unique Hypo-METRICS items. (DOCX)

S2 Table Model fit indices for the morning check-in. (DOCX)

S3 Table Fit indices for the morning, afternoon and evening Hypo-METRICS check-ins. (DOCX)

S4 Table Validity of the Hypo-METRICS work and productivity scores. (DOCX)

S1 Text Statistical analysis (detailed plan). (DOCX)
