# Supplementary material for: Psychometric properties of an innovative smartphone application to investigate the daily impact of hypoglycemia in people with type 1 or type 2 diabetes: The Hypo-METRICS app
Source: PLoS One. 2023 Mar 17;18(3):e0283148. doi: 10.1371/journal.pone.0283148 (PMC10022775; doi:10.1371/journal.pone.0283148)
Supplement: S1 Table — (DOCX) [file pone.0283148.s001.docx]

## **Supplementary S1 table:**

| **S1 Table: The original conceptual framework of the 29 unique Hypo-METRICS items [1]** | | | | |
| --- | --- | --- | --- | --- |
| **Module names and items** | **Conceptual framework domain** | **Completion timepoints (‘Check-ins’)** | | |
|  |  | **Morning** | **Afternoon** | **Evening** |
| ***Sleep quality module (2 items)*** | | | | |
| 1. *How well did you sleep?* | *Sleep quality* | *x* |  |  |
| 2. *When you woke up how did you feel?* | *Sleep quality* | *x* |  |  |
| ***General well-being module (7 items)*** | | | | |
| 3. *How is your mood right now?* | *Mood* | *x* | *x* | *x* |
| 4. *How anxious do you feel right now?* | *Anxiety* | *x* | *x* | *x* |
| 5. *How is your energy level right now?* | *Energy levels* | *x* | *x* | *x* |
| 6. *How irritable do you feel right now?* | *Mood* | *x* | *x* | *x* |
| 7. *How alert do you feel right now?* | *Cognitive function* | *x* | *x* | *x* |
| 8. *How easy was if for you to remember things today?* | *Cognitive function* |  |  | *x* |
| 9. *How well are you able to concentrate right now?* | *Cognitive function* | *x* | *x* | *x* |
| ***Fear of hypo-/hyperglycaemia module (4 items)*** | | | | |
| *10. How worried are you about having a hypo later today?* | *Fear* | *x* | *x* |  |
| *11. How worried are you about having high blood glucose later today?* | *Fear* | *x* | *x* |  |
| *12. How worried are you about having a hypo while asleep?* | *Fear* |  |  | *x* |
| *13. How worried are you about having high blood glucose while asleep?* | *Fear* |  |  | *x* |
| ***Social interactions module (1 item)*** | | | | |
| *14. How well did you get along with other people today?* | *Social functioning* |  |  | *x* |
| ***Work and productivity module (4 items)*** | | | | |
| *15. How many hours did you work today?* | *Work/productivity* |  |  | *x* |
| *16. How many hours did you miss from work for ANY reason today? [this includes health issues, vacation, holiday, etc.]* | *Work/productivity* |  |  | *x* |
| *17. How many hours did you miss from activities other than work today for ANY reason (e.g. study, housework, shopping, family or leisure activities)?* | *Leisure activities* |  |  | *x* |
| *18. How productive were you while working today? (* | *Work/productivity* |  |  | *x* |
| ***Self-report of hypos while asleep module* (8 items)*** | | | | |
| *19. During the night, did you have a hypo OR take action to prevent a hypo that was about to happen?*** | *NA* | *x* |  |  |
| *20. How many hypos did you have?* | *NA* | *x* |  |  |
| *21. At what time did this happen?* | *NA* | *x* |  |  |
| *22. How did you detect your hypo or a hypo that was about to happen? (Select all that apply)* | *NA* | *x* |  |  |
| *23. What happened? (Select all that apply)* | *NA* | *x* |  |  |
| *24. Overall: How bothersome was hypoglycaemia for you last night?* | *Burden* | *x* |  |  |
| *25. Overall: How much sleep did you lose due to hypoglycaemia?* | *Sleep quality* | *x* |  |  |
| *26. Overall: How worried were you about going back to sleep?* | *Sleep quality* | *x* |  |  |
| ***Self-report of daytime hypos module* (7 items)*** | | | | |
| *27. Today, did you have a hypo OR take action to prevent a hypo that was about to happen?*** | *NA* |  |  | *x* |
| *20.1 How many hypos did you have?* | *NA* |  |  | *x* |
| *21.1 At what time did this happen?* | *NA* |  |  | *x* |
| *22.1 How did you detect your hypo or a hypo that was about to happen?* | *NA* |  |  | *x* |
| *23.1 What happened?* | *NA* |  |  | *x* |
| *28. Overall: How bothersome was hypoglycaemia for you today?* | *Burden* |  |  | *x* |
| *29. Overall: How long was it before you were feeling your "usual self" again?* | *Daily living / usual activities* |  |  | *x* |

1. Søholm, U., et al., *Investigating the day-to-day impact of hypoglycaemia in adults with type 1 or type 2 diabetes: design and validation protocol of the Hypo-METRICS application.* BMJ Open, 2022. **12**(2): p. e051651.
