# Supplementary material for: Psychometric properties of an innovative smartphone application to investigate the daily impact of hypoglycemia in people with type 1 or type 2 diabetes: The Hypo-METRICS app
Source: PLoS One. 2023 Mar 17;18(3):e0283148. doi: 10.1371/journal.pone.0283148 (PMC10022775; doi:10.1371/journal.pone.0283148)
Supplement: S2 Table — (DOCX) [file pone.0283148.s002.docx]

## **Supplementary S2 Table**

| S2 Table: Model fit indices for the morning check-in | | | | |  |  |
| --- | --- | --- | --- | --- | --- | --- |
|  | | | | |  |  |
|  |  | 1) Level one model | 2) Null model | 3) Independence model | 4) Saturated model | 5) Hypothesized model |
|  | **χ2** | 568.664 | 44014.806 | 1798.238 | 568.721 | 767.624 |
|  | **df** | 22 | 77 | 67 | 22 | 44 |
|  | **CFI** | 0.972 | 0.000 | 0.918 | 0.974 | 0.966 |
|  | **TLI** | 0.944 | -1.446 | 0.889 | 0.893 | 0.930 |
|  | **RMSEA** | 0.063 | 0.301 | 0.064 | 0.063 | 0.051 |
|  | **SRMR (within)** | 0.031 | 0.189 | 0.031 | 0.031 | 0.031 |
|  | **SRMR (between)** | NA | NA | 0.579 | 0.001 | 0.084 |
|  | **AIC** | 171744.147 | 221823.619 | 179627.051 | 178487.534 | 178642.437 |
| Procedure by Huang (built on recommendations from Hox) [1] :  1: Level one model: See if there is an adequate fit on the within covariance matrix; if not it might not make sense to continue with a multilevel CFA.  2: Null model: A poor fit for this model indicates that there is between-group variance (i.e., a good model fit would indicate no group-level variance).  3: Independence model: If this model fits well this indicates group-level variance but no interesting structural model and vice versa; bad fit indicates that there is a structural model at the group level which should be modelled.  4: Saturated model: Poor model fit here would indicate an error was made or that the model specified in step 1 did actually do not provide good model fit.  5: Hypothesized model: test the hypothesized model and assess model fit  Conclusion from the above 5 step-procedure is that a multilevel factor analysis seems appropriate.  χ2: Chi-square test statistic (in Lavaan found under ‘Test Statistic’ for the ‘Model Test User Model’)  df: Degrees of freedom  CFI: Comparative Fit Index  TLI: Tucker Lewis index  RMSEA: Root-Mean-Square Error of Approximation  SRMR: Standardized Root-Mean-square Residual  AIC: Akaike information criterion | | | | | | |

1. Huang, F., *Conducting Multilevel Confirmatory Factor Analysis Using R*. 2017.
