# Supplementary material for: Psychometric properties of an innovative smartphone application to investigate the daily impact of hypoglycemia in people with type 1 or type 2 diabetes: The Hypo-METRICS app
Source: PLoS One. 2023 Mar 17;18(3):e0283148. doi: 10.1371/journal.pone.0283148 (PMC10022775; doi:10.1371/journal.pone.0283148)
Supplement: S3 Table — (DOCX) [file pone.0283148.s003.docx]

| S3 Table: Fit indices for the morning, afternoon and evening Hypo-METRICS check-ins | | | | | | | | | |
| --- | --- | --- | --- | --- | --- | --- | --- | --- | --- |
|  | **Morning check-in** | | | **Afternoon check-in** | | | **Evening check-in** | | |
| Model: | **A** | **B** | **C** | **A** | **B** | **C** | **A** | **B** | **C** |
| χ2 | 422.922 | 211.212 | 341.671 | 279.827 | 175.137 | 141.999 | 370.279 | 230.609 | 205.581 |
| df | 44 | 44 | 36 | 24 | 24 | 18 | 36 | 36 | 26 |
| Robust CFI | 0.967 | 0.985 | 0.972 | 0.959 | 0.976 | 0.979 | 0.952 | 0.972 | 0.973 |
| Robust TLI | 0.932 | 0.969 | 0.929 | 0.905 | 0.944 | 0.934 | 0.879 | 0.931 | 0.907 |
| Robust RMSEA | 0.050 | 0.034 | 0.051 | 0.058 | 0.044 | 0.048 | 0.054 | 0.041 | 0.053 |
| SRMR (within) | 0.031 | 0.019 | 0.031 | 0.032 | 0.026 | 0.023 | 0.030 | 0.023 | 0.022 |
| SRMR (between) | 0.084 | 0.041 | 0.031 | 0.082 | 0.034 | 0.027 | 0.074 | 0.040 | 0.033 |
| AIC | 178642.437 | 178267.563 | 178546.683 | 145361.268 | 145153.179 | 145123.928 | 187310.677 | 187020.840 | 187012.304 |
| χ2: Chi-square test statistic (in Lavaan found under ‘Test Statistic’ for the ‘Model Test User Model’)  df: Degrees of freedom  Robust CFI: Robust Comparative Fit Index  Robust TLI: Robus Tucker Lewis index  Robust RMSEA: Robust Root-Mean-Square Error of Approximation  SRMR: Standardized Root-Mean-square Residual  AIC: Akaike information criterion  Model A: the theoretical factor structure based on previous conceptual framework.  Model B: the first adjusted model with “How irritable do you feel right now?” paired with “How anxious do you feel right now?”.  Model C: the second adjusted model with fear of hypoglycaemia and fear of hyperglycaemia items separated. | | | | | | | | | |

## **Supplementary S3 table:**
