# Supplementary material for: Psychometric properties of an innovative smartphone application to investigate the daily impact of hypoglycemia in people with type 1 or type 2 diabetes: The Hypo-METRICS app
Source: PLoS One. 2023 Mar 17;18(3):e0283148. doi: 10.1371/journal.pone.0283148 (PMC10022775; doi:10.1371/journal.pone.0283148)
Supplement: S4 Table — (DOCX) [file pone.0283148.s004.docx]

## **Supplementary S4 table**

| S4 Table: Validity of the Hypo-METRICS work and productivity scores | | | | |
| --- | --- | --- | --- | --- |
|  |  | **Summed and mean values (week 3)** | |  |
| Hypo-METRICS work and productivity items (n=60) | **WPAI equivalent items (n=50)** | **Hypo-METRICS app** | **WPAI:SHP** | **Convergent validity*** |
| How many hours did you work today?  Approximately:  ___ Hours ____ Minutes | (4) During the past seven days, how many hours did you actually work?  _____ HOURS | 26.02 ± 14.13 | 29.88 ± 14.17 | r_s_=0.72 |
| How many hours did you miss from work for ANY reason today? [this includes health issues, vacation, holiday, etc.]  Approximately:  ___ Hours ____ Minutes | (2) During the past seven days, how many hours did you miss from work because of problems associated with your hypoglycaemia?  _____ HOURS  PLUS  (3) During the past seven days, how many hours did you miss from work because of any other reason, such as vacation, holidays, time off to participate in this study?  _____HOURS | 2.12 ± 5.70 | 3.87 ± 8.56 | r_s_=0.18 |
| How many hours did you miss from activities other than work today for ANY reason (e.g. study, housework, shopping, family or leisure activities)?  Approximately:  ___ Hours ____ Minutes | (6) During the past seven days, how much did your PROBLEM affect your ability to do your regular daily activities, other than work at a job?  Hypoglycaemia had no effect on my daily activities (0) - Hypoglycaemia completely prevented me from doing my daily activities (10). | 1.26 ± 3.74 | 2.61 ± 2.21 | r_s_=0.03 |
| How productive were you while working today?  Extremely unproductive (0) – Extremely productive (10) | (5) During the past seven days, how much did your hypoglyceamia affect your productivity while you were working?  Hypoglycaemia had no effect on my work (0) - Hypoglycaemia completely prevented me from working (10) | 7.10 ± 1.55* | 2.02 ± 2.05 | r_s_=-0.39 |
| All correlations were a priori hypothesized to be moderate (Spearman rs > ±0.3).  *not summed across the week 3, but just averaged.  **Spearman’s rho correlations | | | | |
