# Supplementary material for: Psychometric properties of an innovative smartphone application to investigate the daily impact of hypoglycemia in people with type 1 or type 2 diabetes: The Hypo-METRICS app
Source: PLoS One. 2023 Mar 17;18(3):e0283148. doi: 10.1371/journal.pone.0283148 (PMC10022775; doi:10.1371/journal.pone.0283148)
Supplement: S1 Text — (DOCX) [file pone.0283148.s005.docx]

## **Supplementary S1 text**

**Statistical analysis (detailed plan):**

Statistical analyses were conducted using R Studio [1]. Descriptive statistics were used to determine sample characteristics, completion rates, distribution of the data, and floor and ceiling effects (i.e., more than 15% of the responses on the highest or lowest possible score [2]). Sample characteristics are presented as number and percentage, or mean ± SD. Completion rates were determined by the percentage of check-ins submitted and the percentage of items skipped in each submitted check-in. In case of non-normality of item responses (explored by skewness and kurtosis values >2.0 [3] and inspection of histograms), robust estimation methods and non-parametric tests (Spearman’s rho r_s_ correlation) were applied. Between person variance in relation to total variance was examined using the Intraclass Correlation Coefficient (ICC) [4] and day-to-day variability in scores was examined using the Root Mean Squared Successive Difference (RMSSD) with values of zero representing no variability (i.e., same responses) across the study period [5]. Inter-item correlation plots were examined to identify items pairs with very low (<0.2) or very high correlations (>0.9) [2]. Multicollinearity was considered to not be a problem when the determinant value is above ≥0.00001 [6]. The Kaiser-Meyer-Olkin (KMO) test of sampling adequacy was used to explore factorability (proportion of common variance) of the items, with values of ≥0.6 suggesting that factor analysis is appropriate [3].

In a first step, structural validity was examined [7]. A multi-level confirmatory factor analysis (MCFA) was conducted, using the previously developed conceptual framework (S1 Table) to structure the model [8]. Item responses were analyzed according to check-in timing: morning, afternoon or evening check-in items. Items were included in the MCFA if they: 1) were asked every day irrespective of whether the participant experienced a hypoglycemic episode, and 2) were not part of the work and productivity module of the app, where most items are relevant only to participants engaged in paid work. With 100 participants and a maximum of 10 unique items per check-in, a 1:10 item to participant ratio was considered acceptable for conducting factor analysis [7]. For some items (Table 3), scores were reversed such that higher scores on all items would indicate “better” daily functioning. The five-step MCFA approach outlined by Huang was first applied on the morning check-in [8], examining the appropriateness of a multilevel analysis by using maximum likelihood estimation to extract variance-covariance data at each level (between- and within-person level) of the data. The approach was modified by using the ‘cluster’ argument in the Lavaan package as presented by Rosseel [9]. The following indices and values were used as indication of good global model fit: comparative fit index (CFI) >0.95, Tucker Lewis index (TLI) >0.95, the Standardized Root-Mean-square Residual (SRMR) <0.08 and Root-Mean-Square Error of Approximation (RMSEA) <0.06 [3, 10]. Standardized factor loadings for each item indicated how much of the response variance in a certain item was explained by the factor, with a desired value >0.7 [3]. Correlation residuals <0.10 indicated good fit, and modification indices were checked to inspect iterations that could improve model fit [3].

Internal consistency reliability of the scales were calculated with use of McDonald’s ω (which has been recommended due to inappropriate assumptions and risk of underestimating reliability using Cronbach’s α [11]). McDonald’s ω scores r_s_>0.7 were considered to indicate satisfactory internal consistency of the scales [11]. Following demonstration of the structural validity and internal consistency, scale scores were calculated by averaging scores on all items within a scale. Test-retest reliability was explored by correlating average scores on each scale from week 3 (test condition) with average scores from week 8 (re-test). Week 3 was selected to allow a two-week run-in phase for participants to familiarize themselves with the app, and week 8 was select to allow for an inter-test interval similar to other studies [12]. Correlations r>0.7 were considered suitable for demonstrating test-retest reliability.

Finally, convergent and divergent validity was investigated by correlating item and factor scores with validated PROMs and participant characteristics. A priori hypotheses were specified relating to convergent and divergent validity (see Table 5 and S4 Table). Convergent validity was supported if Spearman correlations (between app scales and PROMs) were strong (r_s_>±0.5) or moderate (r_s_>±0.3), and divergent validity was supported if they were low (r_s_<±0.3) [13]. ‘Financial situation (DIDP question)’, ‘HbA1C (mmol/mol)’ and ‘Diabetes duration’ (Table 5) were included for exploring divergent validity. Given that PROMs typically require respondents to reflect over a given period of time (e.g., ‘past seven days’), daily app scores were averaged over a period of time corresponding to the PROM’s recall period. For example, if the PROM’s recall period was the ‘past seven days’, then the corresponding app scores were averaged across the final seven days of the study period and correlated with the PROM score. Analysis of convergent validity for the work and productivity related items was also conducted on the subsample for whom those items were applicable (S4 Table).

1. Team, R. *RStudio: Integrated Development Environment for R.* RStudio, PBC, Boston, MA 31-08-2021]; Version 1.4.1717:[Available from: <http://www.rstudio.com/>.

2. Vet, H.C.W.d., et al., *Measurement in Medicine*. 2011, Cambridge: Cambridge University Press.

3. Knekta, E., C. Runyon, and S. Eddy, *One Size Doesn't Fit All: Using Factor Analysis to Gather Validity Evidence When Using Surveys in Your Research.* CBE life sciences education, 2019. **18**(1): p. rm1-rm1.

4. Theobald, E., *Students Are Rarely Independent: When, Why, and How to Use Random Effects in Discipline-Based Education Research.* CBE Life Sci Educ, 2018. **17**(3): p. rm2.

5. Woyshville, M.J., et al., *On the meaning and measurement of affective instability: clues from chaos theory.* Biol Psychiatry, 1999. **45**(3): p. 261-9.

6. Field, A., J. Miles, and Z. Field, *Discovering Statistics Using R*. 2012: Sage Publications Ltd.

7. Terwee, C., et al., *Quality criteria were proposed for measurement properties of health status questionnaires.* Journal of clinical epidemiology, 2007. **60**: p. 34-42.

8. Huang, F., *Conducting Multilevel Confirmatory Factor Analysis Using R*. 2017.

9. Rosseel, Y. *Multilevel Structural Equation Modeling with lavaan*. 2020 [cited 2021 August 2nd]; Available from: <https://users.ugent.be/~yrosseel/lavaan/zurich2020/lavaan_multilevel_zurich2020.pdf>.

10. Mokkink, L.B., et al. *COSMIN methodology for systematic reviews of patient-reported outcome measures (PROMs)—user manual.* 2018 [cited 2021 28 April]; Available from: <https://www.cosmin.nl/> wp-content/uploads/COSMIN-syst-review-for-PROMs-manual_ version-1_feb-2018.pdf.

11. McNeish, D., *Thanks coefficient alpha, we'll take it from here.* Psychol Methods, 2018. **23**(3): p. 412-433.

12. Granholm, E., et al., *What Do People With Schizophrenia Do All Day? Ecological Momentary Assessment of Real-World Functioning in Schizophrenia.* Schizophr Bull, 2020. **46**(2): p. 242-251.

13. Cohen, J., *A power primer.* Psychol Bull, 1992. **112**(1): p. 155-9.
